# Impact of metabolic and bariatric surgery on the paediatric & adolescent metabolome: A systematic review and meta-analysis

**DOI:** 10.1038/s41598-025-20078-7

**Published:** 2025-10-16

**Authors:** Anuja T. Mitra, Olivia Wing, Bibek Das, Naim Fakih- Gomez, Haris Khwaja, Chetan Parmar, Matyas Fehervari

**Affiliations:** 1 Department of Surgery and Cancer, Imperial College London, Hammersmith Hospital, Du Cane Road, 7NB Commonwealth Building, London, W12 ONN UK; 2 Imperial College School of Medicine, Imperial College London, London, W12 0NN UK; 3 Chelsea & Westminster Hospital, NHS Foundation Trust, 369 Fulham Rd, London, SW10 9NH UK; 4 Department of Targeted Intervention, University College London, Gower Street, London, WC1E 6BT UK; 5 Whittington Hospital, London, N19 5NF UK; 6 Tunbridge Wells Hospital, Pembury, Tunbridge Wells, TN2 4QJ UK; 7 Kent & Medway Medial School, University of Kent & University of Canterbury Christchurch, Canterbury, UK

**Keywords:** Childhood obesity, Metabolic bariatric surgery, Metabolomic, Weight loss, Molecular biology, Systems biology, Diseases, Medical research

## Abstract

Metabolic bariatric surgery (MBS) is an effective treatment for paediatric obesity, yet the mechanisms underlying weight loss remain unclear. This systematic review and meta-analysis evaluated the short- and long-term effects of MBS on the paediatric metabolome to provide insights into metabolic pathways contributing to surgical outcomes. This prospectively registered systematic review (PROSPERO ID: CRD42024607784) adhered to PRISMA guidelines. Meta-analysis was undertaken on pre-defined post-operative weight and metabolic parameters in paediatric patients (aged 5- 19 years) following MBS. Outcomes were reported as weighted or standardised mean Difference with 95 percent confidence intervals from random effects modelling. Quality scoring and quantitative assessment of bias were performed. Results from 12 studies (451 patients, mean age 16.9 years) across five countries were included. The median follow-up was 12 months. Patients underwent Roux-en-Y gastric bypass (RYGB, n = 275) or laparoscopic sleeve gastrectomy (LSG, n = 140). Most studies used serum and urine assays; two included tissue biosamples. MBS was associated with significant long-term weight reduction, with a mean BMI decrease of -14.4 kg/m^2^ (95% CI: -17.5 to -11.3) and %TWL of 25% (95% CI: 18.6 to 32.2). Metabolic improvements included reduced cholesterol (-10 mg/dL), LDL (-14.6 mg/dL), triglycerides (-33.3 mg/dL), and increased HDL (+ 8.0 mg/dL). Significant enhancements were noted in glycaemic, pancreatic and insulin regulation, evidenced by decreased HOMA-IR (-4.1) and C-peptide (-1.8 ng/mL). Liver function parameters, ALT (-14.4 U/L), AST (-5.4 U/L), and GGT (-9.6 U/L) and inflammatory cytokines, IL-6 (-12.2 pg/mL) and TNF-α (-54 pg/mL) significantly declined following surgery. These findings demonstrate a distinct metabolic signature of MBS in adolescents, leading to substantial weight loss and improvements in cardiovascular, glycaemic, and liver health, alongside reduced systemic inflammation. These results underscore the efficacy of MBS as a therapeutic intervention for adolescents living with severe obesity, demonstrating a profound impact on the paediatric metabolome.

**Supplementary Information:**

The online version contains supplementary material available at 10.1038/s41598-025-20078-7.

## Introduction

Obesity has emerged as a global epidemic with profound and multifaceted impacts on physical, mental, and psychosocial health. Once considered primarily a Disease of adulthood, obesity now affects an estimated 160 million children worldwide and is projected to become the fastest-growing global health challenge among young people^1,2^. The consequences of obesity in this cohort may be particularly severe, as it coincides with critical periods of growth and development, including metabolic, endocrine, and bone maturation, all of which can be adversely affected by the pro-inflammatory state associated with obesity^3^. Additionally, the well-documented negative effects of obesity on psychosocial well-being and mental health further exacerbate its burden. As a result, health-related quality of life is significantly diminished in children living with obesity^4^. There is a well-established positive correlation between childhood and adolescent obesity persisting into adulthood. Children and adolescents who have obesity are five times more Likely to remain obese as adults compared to their peers who Do not have obesity. Approximately 55% of children with obesity continue to have obesity in adolescence, and nearly 80% of adolescents living with obesity remain obese in adulthood^5^. This underscores the critical need for early intervention and prevention strategies to curb obesity and its long-term health consequences.

Metabolic Bariatric surgery (MBS) is a well-established and effective treatment for obesity and its associated medical comorbidities^6^. Early intervention in obesity management is widely recognised as beneficial, with evidence suggesting that achieving significant weight loss before adulthood substantially reduces the risk of long-term complications associated with obesity^7,8^. Addressing childhood obesity has become a critical target for intervention and consequently this has been reflected by the increased incidence of MBS in paediatric and adolescent populations in recent years^9,10^. Currently, the American Society for Metabolic and Bariatric Surgery (ASMBS) recommends surgical intervention for children at Tanner pubertal stages 3–4 and beyond with obesity accompanied by cardiometabolic comorbidities^11^. Among the various surgical options, laparoscopic sleeve gastrectomy (LSG) is the most commonly performed procedure in this population.

In the adult population, research efforts have been Directed to explore the impact of MBS on the metabolome to elucidate the mechanisms underlying weight loss. Key metabolic changes identified in adults include early alterations in amino acid and peptide metabolites, shifts in fatty acid and bile acid profiles, and increased levels of ketone bodies such as 3-hydroxybutyrate and acetoacetate^12–14^. Additionally, MBS has been shown to influence gut microbiota, leading to enhanced amino acid and carnitine metabolism post-surgery^15–18^. These studies have provided valuable insights into the metabolic pathways driving weight loss and have opened opportunities for targeted therapies. Due to the nascent nature of paediatric MBS, similar research in this field is limited. Investigating these mechanisms in children is crucial, as it not only deepens our understanding of the effectiveness of MBS but may also inform the development of further anti-obesity treatments, whether medical or surgical. Furthermore, studying paediatric metabolomics could help identify novel mechanistic pathways, refine patient counselling, and provide a clearer understanding of the short- and long-term effects of MBS during this critical developmental period. Consequently, this systematic review and meta- analysis aims to evaluate the impact of MBS on the paediatric and adolescent metabolome and what biological systems it has the greatest impact on to confer profound weight loss.

## Methods

The review was prospectively registered on PROSPERO (CRD42024607784). A comprehensive literature search was performed in accordance with the recommendations of the preferred reporting items for systematic reviews and meta-analysis guidelines (PRISMA) to identify scientific publications reporting any metabolic outcome evaluated using mass spectrometry techniques in patients under the age of 18 years who underwent MBS. Searched databases included MEDLINE (1946 to 10 November 2024), EMBASE (1947 to 10 November 2024) via the Ovid platform and the Cochrane Review Library. Reference lists of eligible articles were also hand-searched for additional publications. The full search strategy can be found in supplementary file 1. All variations in the spelling, including truncated search terms using wild card characters and the “related articles” function, were used in combination with the Boolean operators AND OR.

## Selection of studies: inclusion and exclusion criteria

Following deduplication of search results, titles and abstracts were screened by two independent reviewers (A.T.M and O.L.) with disagreements in study inclusion resolved by a third independent reviewer (B.D). The inclusion criteria required articles to report pre- and post- MBS metabolic outcomes and anthropometric parameters in paediatric/adolescent patients as defined by ASMBS and International Federation for the Surgery of Obesity and Metabolic Disorders (IFSO) guidelines (5- 19 years old) (10). Comparative cohort studies, non-randomised prospective studies, and randomised controlled trials (RCT) were included. Case series, case reports, narrative reviews, editorials, and conference abstracts; or studies with fewer than five participants and publications in a non-English language were excluded.

## Data extraction and quality assessment

Information extracted included year of publication, study design, sample size, country of study, length of follow up, baseline demographics of patients, type of bariatric intervention, pre- and post- operative anthropometric data (weight, body mass index [BMI]) and the following metabolic outcomes; lipid panels (high density lipoprotein [HDL], low density lipoprotein [LDL], triglycerides (TGs) and cholesterol), glucose regulatory markers (glucose, insulin, HbA1c, homeostatic model assessment of insulin resistance [HOMA- IR], glucagon-like peptide-1 [GLP-1] and C-peptide, liver function (alanine aminotransferase [ALT], aspartate aminotransferase [AST], gamma-glutamyl transferase [GGT], and inflammatory cytokine panel (interleukin-1b [IL 1b], interleukin-6 [IL-6] and tumour necrosis factor alpha [TNFα].

All studies were appraised for quality and rigorousness using the Newcastle–Ottawa Scale (NOS) which is a quality assessment tool for non-randomised studies, including case–control and cohort studies, evaluating selection, comparability, and outcome/exposure criteria. It ranges from 0 to 9, with a maximum score of 9 indicating the highest quality (18).

## Outcome measures

All Data was extracted up to the longest follow up. Data was pooled for effect estimates for meta-analysis if there were 2 or more studies. Baseline characteristics are reported as mean values with standard Deviations and percentages. Modulation in outcomes between pre- and post- surgery groups are reported as standardised or weighted mean differences, with 95% confidence intervals.

## Statistical methods

Data analysis was performed using Stata Software, Version 15.1. StataCorp LCC, TX. Random effects analysis was used to calculate weighted mean Difference and mass effect. All studies were included in the analysis if relevant data was available, and results were pooled if 2 or more studies reported an outcome. Groups were dichotomised into ‘short’ (< 6 months) or ‘moderate’ (> 6 month) follow up. Descriptive statistics was described as mean and standard error of the mean. Data for the meta-analysis was analysed using a random effects model and statistical heterogeneity was calculated using I^2^. An I^2^ of < 30 was considered as low, 30–60 as moderate and > 60 as high heterogeneity. Results were computed and represented on forest plots with meta-analyses. Funnel plots with Egger’s test P values were constructed to quantitatively evaluate publication bias.

## Results

### Summary of studies

**Fig. 1:**
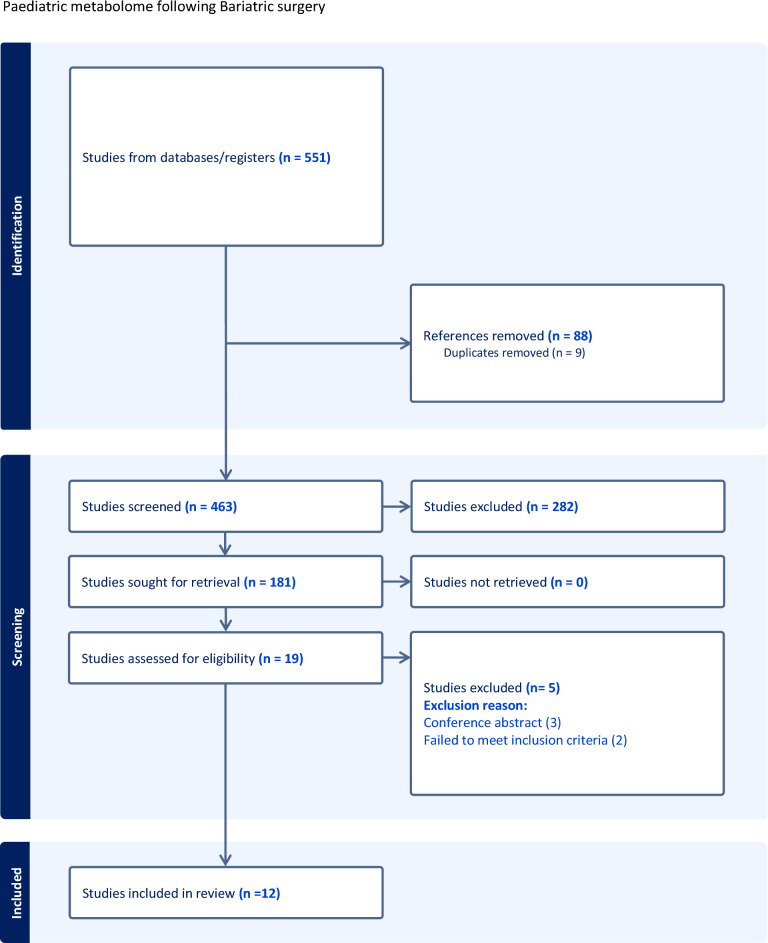
PRISMA flowchart. The flowchart depicts the number of records identified through database searching and other sources, the number of duplicates removed, records screened, full-text articles assessed for eligibility, and studies included in the qualitative and quantitative synthesis.

Our review of the Literature identified 181 studies were retrieved for full-text review based on abstract screening. Of these, 162 were excluded following full-text evaluation. The main reasons for exclusion were Due to the absence of relevant metabolomic or biochemical endpoints or the use of non-mass spectrometry-based platforms. Others reported only anthropometric outcomes without sufficient metabolic data. Nineteen studies, of which 12 met the inclusion criteria were used for the final meta-analysis (see PRISMA flowchart, Fig. 1). Among these, 9 studies were prospective cohort studies, and 3 were retrospective; no RCTs were included. The total number of participants was 415, with 65% identified as female. The mean age of participants was 16.9 years at enrolment, with the youngest participant enrolled at 13 years. Seven studies included a non-operative cohort for comparison, using either peers with obesity (n = 4), lean peers (n = 2), or both (n = 1). Patients underwent either Roux-en-Y gastric bypass (RYGB; n = 275) or laparoscopic sleeve gastrectomy (LSG; n = 140). The median follow-up period was 12 months, with the longest follow-up extending to 96 months post-surgery. Most studies used serum (n = 11) for the biological assay for metabolomic analysis, with 5 also analysing urine and 2 studies using tissue biopsies. In terms of analytical methods and platforms used, three studies used high performance liquid chromatography mass spectrometry (HP-LC/MS) to measure outcomes, most articles used a combination of techniques such as ELISA panels (n = 6) in addition to routine biochemical laboratory assays (n = 5) and immunohistochemistry (n = 2). Of the studies that utilised HP-LC/MS, quality control steps were incorporated to ensure accurate and reliable functioning of the platforms. In terms of quality assessment, NOS reporting standards of the studies ranged from 6 to 9, with the median score being 7.5, rating the included studies as high quality. Study heterogeneity as reflected by the I^2^ statistic ranged from 0- 97%. Study characteristics, patient demographic data and quality assessment scores can be found in Table 1.

**Table 1:** Study characteristics and patient demographics.

First author	Year of publication	Country	Sample size	Mean age at enrolment (years)	Sex (% male)	Type of bariatric intervention	Biological assay for analysis	NOS*
Inge^19^	2019	USA	161	17.0	21.7%	RYGB	serum	9
Pastore^20^	2021	Italy	24	15.3	50.0%	LSG	serum, urine, tissue	8
Shah^21^	2017	USA	58	17.1	36.2%	RYGB	serum	8
Shehata^22^	2021	Egypt	36	16.8	22.2%	LSG	serum	7
Oberbach^23^	2014	Germany	10	15.6	30.0%	LSG & RYGB	serum	8
Davis^24^	2018	USA	10	16.9	not specified	RYGB & LSG	serum, urine	7
Sinha^25^	2013	USA	24	19.0^a^	16.7%	RYGB	serum	8
DeFoor^26^	2016	USA	17	18.2^a^	94.1%	RYGB & LSG	urine	7
Butte^27^	2015	USA	11	16.5	27.3%	RYGB	serum, urine	8
Xiao^28^	2015	USA	22	16.5^a^	77.3%	RYGB & LSG	serum, urine	6
Franco^29^	2017	Brazil	22	16.9	72.7%	LSG	serum	7
Nobili^30^	2018	Italy	20	16.7	35.0%	LSG	serum, tissue	6

^a^Median.

*NOS, Newcastle Ottowa Score.

Role of funding source: This research did not receive any specific grant from funding agencies in the public, commercial, or not-for-profit sectors.

### Weight loss

**Fig. 2:**
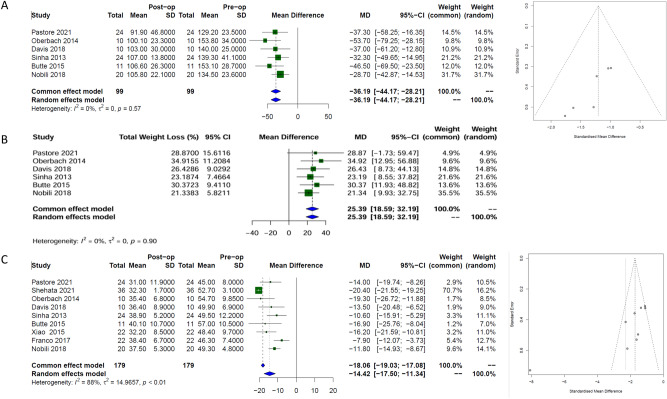
Forest plots with corresponding funnel plots from random-effects modelling, illustrating the long-term effects of bariatric surgery in paediatric patients (n = 99). The forest plots show the estimated effect size for (**A**) absolute weight (kg), (**B**) percentage total weight loss (%TWL), and (**C**) body mass index (BMI, kg/m^2^), while the funnel plots assess potential publication bias.

Six studies, with a total of 99 patients, determined weight loss before and after MBS in paediatric patients. When all bariatric surgeries were pooled, there was an absolute weighted mean reduction of −33.4 kg [95% CI −47.6; −19.3 kg, *I*^*2*^ 0%] in the short term which was maintained in the long term, −36.2 kg [95% CI −44.2; −28.2, *I*^*2*^ 0%)], Fig. 2a. This translated to total weight loss (TWL) of 25% in the long term [95% CI 18.6; 32.2, *I*^*2*^ 0%)], Fig. 2b. Using random effects modelling and Data from nine studies with a total of 179 patients, this translated to a BMI reduction of −12.1 kg/m^2^ [95% CI −14.2; −10.1, *I*^*2*^ 0%] and −14.4 kg/m^2^ [95% CI −17.5; −11.3], *I*^*2*^ 88% in the short and long term, respectively, Fig. 2c. Eggers test result suggested the presence of publication bias in the long term, p = 0.02.

### Glucose and insulin regulation

#### Glucose

**Fig. 3:**
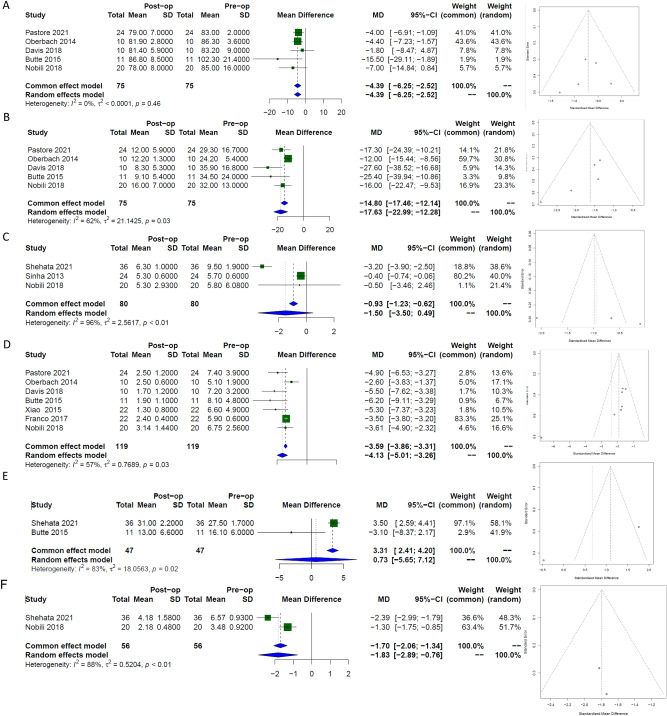
Forest plots with corresponding funnel plots of random effects modelling demonstrating the strength of effect and publication bias respectively from long term data in glucose and insulin parameters in paediatric patients following bariatric surgery. (**A**) glucose, (**B**) insulin, (**C**) HbA1c, (**D**) HOMA-IR, (**E**) GLP1 and (**F**) Connecting peptide.

Raw serum glucose values were evaluated in five papers with a total of 75 patients demonstrating a significant reduction following MBS in the long term, [−4.4 mg/ml, (95% CI −6.3; −2.5)], *I*^*2*^ 0%)], Fig. 3a. Egger’s regression test indicated no significant evidence of publication bias (p = 0.6).

#### Insulin

Five papers evaluated changes in insulin following MBS from a total of 75 patients demonstrating a significant reduction in the long term, [−17.6 units, (95% CI −23.0; −12.3)], *I*^*2*^ 62%)], Fig. 3b. Egger’s regression test indicated no significant evidence of publication bias (p = 0.05).

#### HbA1c

Two papers with 60 patients evaluated short-term changes in HbA1c and three papers with 80 patients evaluated the long- term changes following MBS. Results demonstrated no difference in the short term, [−1.53%, (95% CI −3.93; 0.77)], *I*^*2*^ 97%)] and although there was a trend for reduction in the long- term, this did not reach significance on random effects modelling, [−1.5%, (95% CI −3.5; 0.5)], *I*^*2*^ 96%)], Fig. 3c. Egger’s regression test indicated no significant evidence of publication bias (p = 0.4).

#### Homeostatic model assessment of insulin resistance (HOMA-IR)

Three papers evaluated changes in HOMA-IR following MBS. The Data included results from 55 patients demonstrating a significant reduction following surgery in the short term, [−5.15 mg/dl, (95% CI −6.79; −3.52)], *I*^*2*^ 0%)] which was also Demonstrated in the long term from 7 studies using 119 patients [−4.1 mg/dl, (95% CI −5.0; −3.3)], *I*^*2*^ 57%)], Fig. 3d. Egger’s test result suggested the presence of publication bias in the long term, p = 0.04.

#### GLP1

The Data from 2 papers with a total of 47 patients evaluated changes in GLP1 following MBS. Results did not detect any change following surgery, [6.3 pmol/L, (95% CI −6.4; 19.1)], *I*^*2*^ 94%)]. This was also Demonstrated in the longer-term using data from 47 patients, [0.7 pmol/L, (95% CI −5.7; 7.12)], *I*^*2*^ 83%)], Fig. 3e.

#### Connecting (C)- peptide

Two papers evaluated changes in C peptide following MBS. The Data included results from 56 patients demonstrating a significant reduction following surgery in the long term, [−1.8 ng/ml, (95% CI −2.9; −0.8)], *I*^*2*^ 88%)], Fig. 3f.

### Liver parameters

**Fig. 4:**
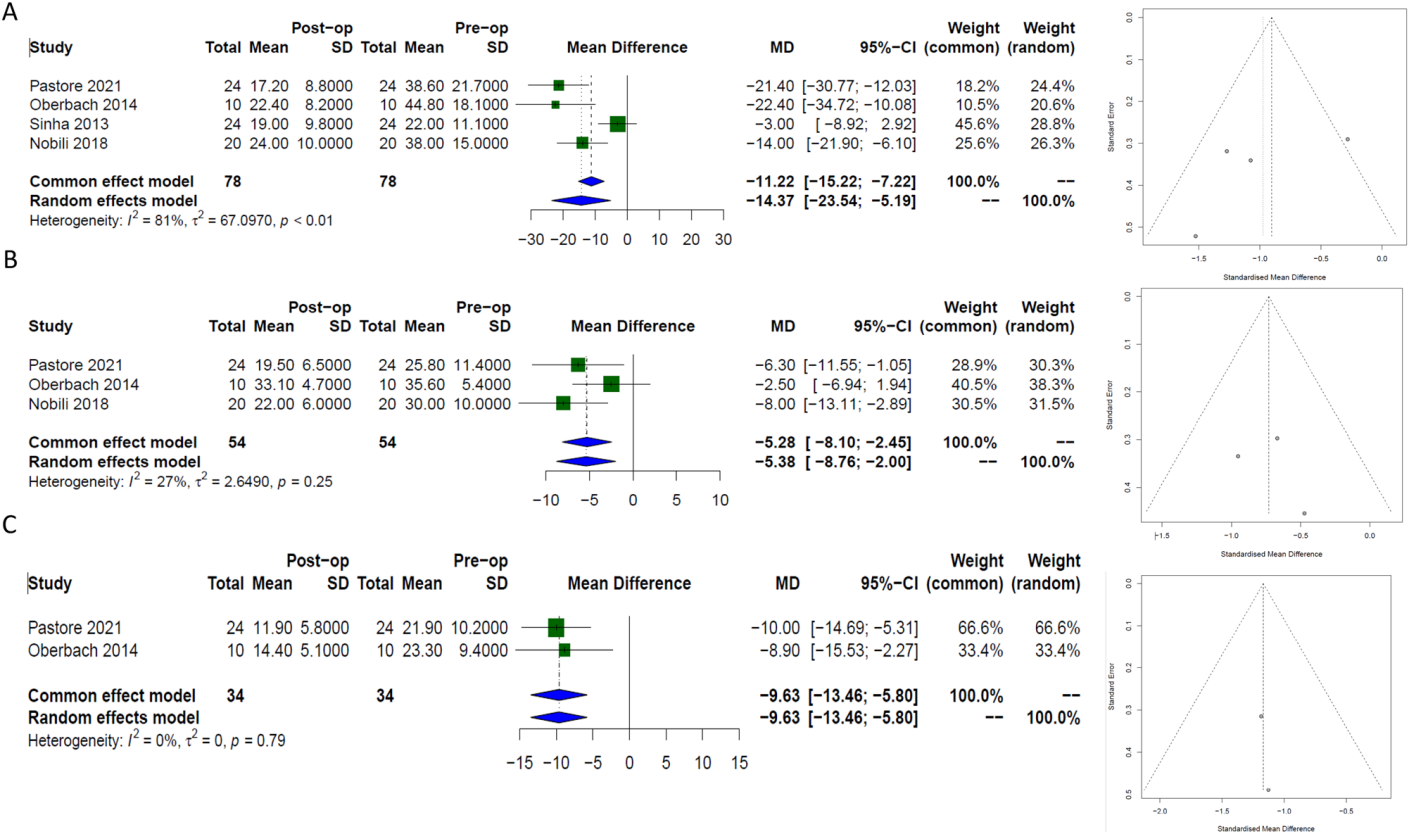
Forest plots with corresponding funnel plots from random-effects modelling, evaluating the long-term effects of bariatric surgery on liver parameters in paediatric patients. The forest plots illustrate the estimated effect size for (**A**) alanine aminotransferase (ALT), (**B**) aspartate aminotransferase (AST), and (**C**) gamma-glutamyl transferase (GGT), while the funnel plots assess potential publication bias.

**ALT**: Four papers evaluated the long- term changes in ALT following MBS. The Data included results from 78 patients demonstrating a significant improvement following surgery, [−14.4 IU/L, (95% CI −23.5; −5.2)], *I*^*2*^ 81%)], Fig. 4a. Egger’s regression test indicated no significant evidence of publication bias (p = 0.3).

**AST**: Three papers evaluated the long- term changes in AST following MBS. The Data included results from 54 patients demonstrating a significant improvement, [−5.4 IU/L, (95% CI −8.8; −2)], *I*^*2*^ 27%)], Fig. 4b. Egger’s regression test indicated no significant evidence of publication bias (p = 0.7).

**GGT**: Two papers evaluated changes in GGT following MBS. The Data included results from 34 patients demonstrating a significant reduction following surgery in the long term, [−9.6 IU/L, (95% CI −13.5; −5.8)], *I*^*2*^ 0%)], Fig. 4c.

### Lipid panel

#### Cholesterol

**Fig. 5:**
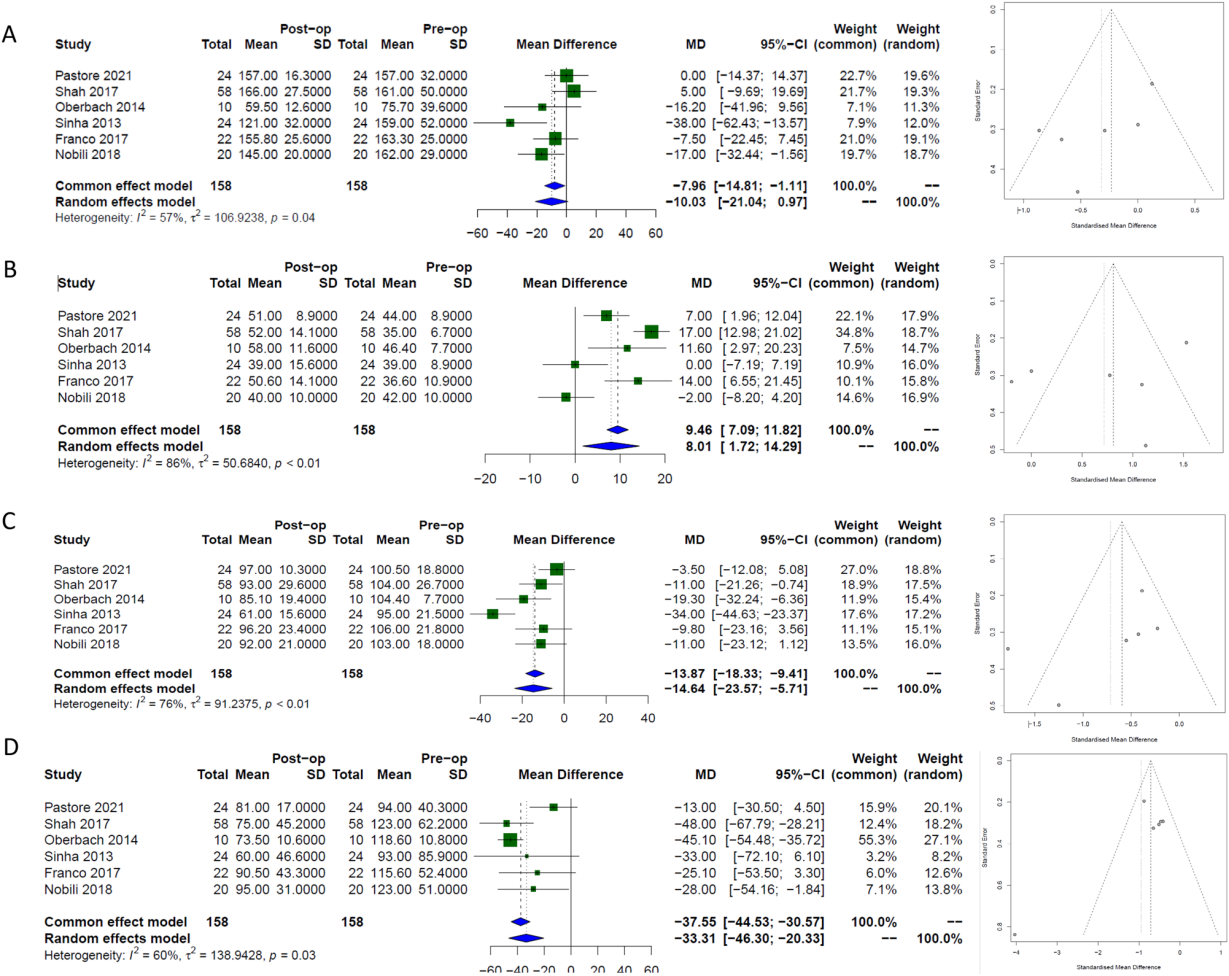
Forest plots with corresponding funnel plots assessing strength of effect and publication bias respectively using random effects modelling of long-term data in lipid panel parameters in paediatric patients following bariatric surgery. (**A**) cholesterol, (**B**) HDL, (**C**) LDL and (**D**) Triglycerides.

Short term cholesterol outcomes were evaluated in two papers with a total of 46 patients. Results Demonstrated a significant reduction at 6 months [−24.8 mg/dl, (95% CI −48.6; −0.9)], *I*^*2*^ 78%)] and beyond using Data from 6 papers with 158 patients, [−10 mg/dl, (95% CI −21; −1.0)], *I*^*2*^ 78%)], Fig. 5a. Egger’s regression test indicated no significant evidence of publication bias (p = 0.1).

#### HDL

Two papers evaluated the short-term outcomes in HDL with a total of 46 patients, and six papers evaluated the long-term outcomes in HDL with a total of 158 patients. Results Demonstrated no significant change in HDL up to 6 months following MBS, [3.03 mg/dl, (95% CI −2.6; 8.6)], *I*^*2*^ 43%)] but a significant increase in the long- term, [8.0 mg/dl, (95% CI 1.7; 14.3)], *I*^*2*^ 86%)], Fig. 5b. Egger’s regression test indicated no significant evidence of publication bias (p = 0.5).

#### LDL

Two papers evaluated the short-term outcomes in LDL with a total of 46 patients demonstrating a significant reduction, [−23.3 mg/dl, (95% CI −45; −1.6)], *I*^*2*^ 88%)]. Six papers evaluated the long-term outcomes in LDL with a total of 158 patients which also demonstrated a significant reduction, [−14.6 mg/dl, (95% CI −23.6; −5.7)], *I*^*2*^ 76%)], Fig. 5c. Egger’s regression test indicated no significant evidence of publication bias (p = 0.22).

#### Triglycerides

The Data from 46 patients were used to evaluate the impact of MBS on TG levels in the short-term demonstrating no significant change following surgery, [1.9 mg/dl, (95% CI −63.9; 1.6)], *I*^*2*^ 90%)]. Six papers evaluated the long-term outcomes with a total of 158 patients demonstrating a significant reduction in TG levels following surgery [−33.3 mg/dl, (95% CI −46.3; −20.3)], *I*^*2*^ 60%)], Fig. 5d. Egger’s regression test indicated no significant evidence of publication bias (p = 0.2).

### Inflammatory markers

#### TNFα

**Fig. 6:**
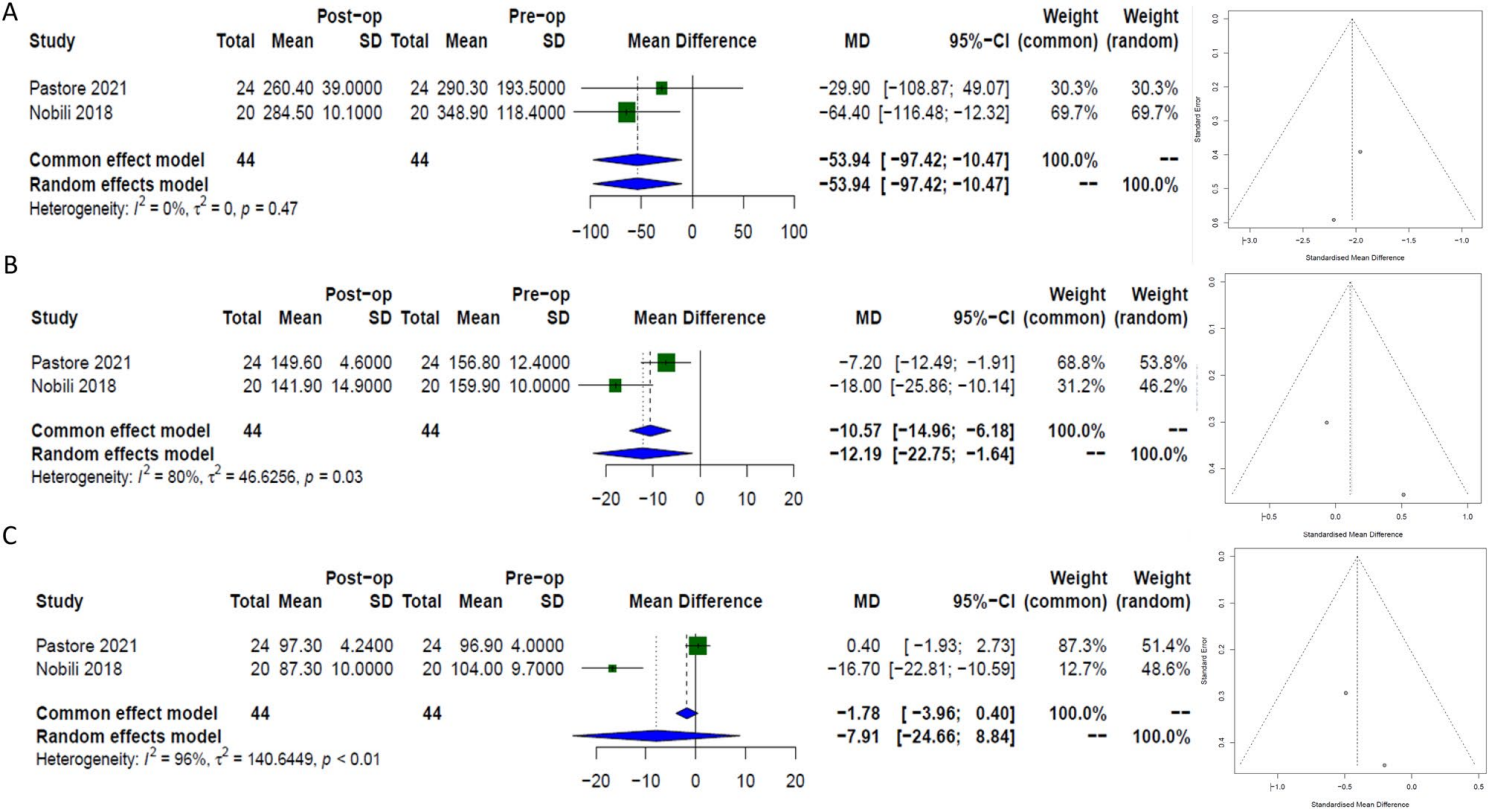
Forest plots with corresponding funnel plots assessing using random effects modelling strength of effect and publication bias respectively from long term data in the following panel of inflammatory cytokines in paediatric patients following bariatric surgery. (**A**) TNFα, (**B**) IL- 6 and (**C**) lL-1β.

Two papers evaluated changes in TNFα following MBS. The Data included results from 44 patients demonstrating a significant reduction following surgery in the long term, [−54 pg/ml, (95% CI −97.4; −10.5)], *I*^*2*^ 0%)], Fig. 6a.

#### IL- 6

Two papers evaluated changes in IL-6 following MBS. The Data included results from 44 patients demonstrating a significant reduction following surgery in the long term, [−12.2 pg/ml, (95% CI −22.8; −1.6)], *I*^*2*^ 80%)], Fig. 6b.

#### IL- 1β

Two papers evaluated changes in lL-1β following MBS. The Data included results from 44 patients demonstrating a non-significant trend towards a reduction following surgery in the long term, [−7.9 pg/ml, (95% CI −24.7; 8.8)], *I*^*2*^ 96%)], Fig. 6c.

## Discussion

To our knowledge, this is the first systematic review and meta-analysis that evaluates the impact of MBS on the paediatric and adolescent metabolome. The seminal findings present evidence for the development of a unique metabolic signature representative of MBS in paediatric patients. The results demonstrate the significant benefits of MBS in this population, including long-term significant weight loss, overall improvements across a range of metabolic markers, and reductions in systemic inflammation.

In adults, the mechanisms behind weight loss and medical co-morbidity resolution through MBS has been shown to occur through cross- talk between weight dependent and independent factors^31^. In children, the impacts of MBS were characterised by significant weight loss in the short and long term with significant alterations in metabolic health. The 25% TWL achieved in adolescents was comparable to the expected outcomes in adults undergoing MBS, which typically ranges from 20–30% TWL for SG and RYGB^32^.

One of the most clinically significant outcomes identified was the consistent improvement in glycaemic control. Reductions in fasting glucose, insulin, C-peptide, and HOMA-IR following MBS suggest enhanced insulin sensitivity and pancreatic function. These changes are crucial given the rising global prevalence of type 2 diabetes mellitus (T2DM) among adolescents^7^. Proposed mechanisms underlying these metabolic improvements include the reorganisation of pancreatic islet cell architecture, which enhances insulin secretion and glucose regulation^33,34^ as well as a combination of mechanisms relating to starvation, ghrelin release and modulation of foregut and hindgut hormones^35–37^. Additionally, the physical reduction in visceral fat mass following bariatric surgery contributes to a decrease in adipokine secretion and the resolution of metabolic perturbations affecting insulin signalling pathways^35^. Together, these effects underscore the multifaceted benefits of bariatric surgery in mitigating T2DM risk in this population.

This review did not demonstrate a significant modulation in GLP-1 levels post-surgery, a finding that contrasts with the widely recognised beneficial role of GLP-1 in mediating the benefits of bariatric procedures. Whilst this may partly reflect the small sample size in the meta-analysis and methodological heterogeneity in sample acquisition, it may also highlight the complexity of hormonal adaptations following MBS in paediatric populations. GLP-1 analogues, such as semaglutide, have shown promise in promoting weight loss and metabolic improvements in children, potentially offering a logical alternative or adjunct to surgery. A double-blind, placebo-controlled RCT by Weghuber et al. involving 201 adolescents Living with obesity Demonstrated significant BMI reductions over 68 weeks with once-weekly injections of semaglutide (− 16.1% vs. − 0.6% with placebo)^38^. However, their use and long- term durability may not be appropriate for the paediatric population. Semaglutide has been associated with a higher incidence of gastrointestinal adverse events (62% vs. 42% compared to placebo) and cholelithiasis in 4% of treated participants, raising concerns about its tolerability and long-term safety. Moreover, Discontinuation rates and adherence to anti-obesity medications may pose significant challenges, particularly in peadiatric populations. For instance, a study of 195,915 adults in the United States using semaglutide reported a 37% Discontinuation rate at 12 months, with higher rates observed among individuals using the medication solely for obesity management rather than diabetes and those with greater social needs^39^. In contrast, bariatric surgery does not face similar adherence issues and offers more comprehensive and sustained benefits, such as durable improvements in glucose homeostasis and liver function, which appear to be independent of GLP-1 pathways, as demonstrated in this study. These findings highlight the potential limitations of pharmacological approaches in peadiatric populations, demonstrating MBS as a potentially more effective, enduring, and safer alternative for managing severe obesity in youth.

There was a significant improvement in liver health amongst children and adolescents following MBS, evidenced by marked reductions in ALT, AST, and GGT. This highlights the potential of MBS to address hepatic complications such as metabolic dysfunction-associated fatty liver disease (MAFLD) linked to paediatric obesity. The benefits of MBS on MAFLD are well-documented in adults and increasingly recognised in paediatric populations^40^. LSG has been shown to induce histological improvements in the Liver of children Living with obesity, as Demonstrated in a study of 20 paediatric patients who underwent LSG^30^. These histological changes are associated with reduced activation of key cellular compartments, including hepatic progenitor cells, hepatic stellate cells, and macrophages, underscoring the importance of cellular interactions and the role of hepatic adipocytokine production in liver regeneration following MBS.

Although our meta-analysis did not directly assess the gut microbiome, emerging literature suggests that it may play a pivotal role in mediating metabolic improvements following MBS, potentially independent of weight loss. Penney et al. demonstrated metabolic improvements in adults with T2DM post-MBS were primarily Driven by changes in the microbiome, with only 4% of pathways overlapping with weight loss-associated mechanisms^18^. Similar shifts have been observed in adolescent cohorts, with Akagbosu et al. reporting increased alpha diversity and enrichment of oral-associated taxa following sleeve gastrectomy^41^. Interestingly, this microbial shift was also accompanied by increased expression of pro-inflammatory genes, demonstrating the complexity of the gut-metabolic axis in younger patients and the potential for age-specific responses to MBS. Further longitudinal and multi-omic studies will be crucial to understanding these interactions.

Systemic inflammation plays a central role in obesity-related comorbidities. In our study, on evaluation of a panel of inflammatory markers, we demonstrated decreases in IL-6 and TNF-α suggesting improvements in the inflammatory milieu and systemic inflammation, a key driver of obesity-related comorbidities. Inflammatory modulation represents another key finding in our review. MBS has been linked to a reduction in inflammation, as evidenced by changes in inflammatory markers. We observed consistent reductions in IL-6 and TNF-α, two cytokines heavily implicated in obesity-related comorbidities. IL-6 a key mediator in the inflammatory response, is secreted by local macrophages at the site of inflammation, where it activates T and B lymphocytes and facilitates the transition from acute to chronic inflammation (10,11). Both adipocytes and adipose tissue macrophages contribute to IL-6 production, and MBS has been shown to lower serum IL-6 levels (24,25). Adipokines, which include adiponectin, leptin, resistin, TNF-α, interleukins, and other biomolecules, influence numerous physiological processes. These molecules play a critical role in regulating appetite, food intake, and body weight (26,27). Pro-inflammatory cytokines such as TNF-α and IL-6 are predominantly produced by visceral adipose tissue (VAT) in the abdomen, emphasising the role of adiposity in systemic inflammation (28). Given their central role in metabolic signalling, it is unsurprising that reductions in adipokine levels are observed in children following MBS. However, the absence of significant changes in IL-1β in our review highlights the complexity of the inflammatory response to MBS, warranting further investigation into these nuanced mechanisms.

There are some limitations to consider when interpreting the results in this study. First, the relatively small sample size which reflects the emerging nature of paediatric MBS research, limits the generalisability of the findings. There is a need for larger, high-quality trials to strengthen the evidence base. As a function of small sample sizes, the heterogeneity between some pooled studies for outcomes of interest were high, which warrants cautious interpretation and may reduce the strength of the conclusions. Although Egger’s tests were employed to assess publication bias, these results are most reliable when Derived from datasets containing more than 10 pooled studies, therefore the reliability of our bias estimates may be compromised. Additionally, the biosamples used across studies were not uniform (e.g., serum, urine, tissue) which presents challenges when standardising outcomes. To improve comparability, future research should aim to use consistent sample types and standardised methodologies. Additionally, it would be important for research to incorporate longer follow-up periods. This would be essential not only to assess the sustained effectiveness of MBS but also to monitor potential complications over time. Assessing long-term changes to the metabolome would provide valuable insights into long-term mechanisms of MBS and any compensatory mechanisms that may develop. It must be acknowledged this data provides an overview of the current metabolic phenotype associated with MBS in adolescents. While adult studies have long demonstrated that MBS exerts its effects through a combination of weight-dependent and weight-independent mechanisms, our study was not designed to distinguish between these and the improvements we observed represent associative post-surgical changes, not causality.

To build upon these findings, future studies should incorporate multi-omic phenotypic profiling using paired samples. This approach would provide a comprehensive understanding of the metabolic and inflammatory pathways influenced by MBS and lead to novel therapies nuanced for paediatric patients for weight loss or co-morbidities remission. Additionally, given the growing interest in the gut microbiome’s role in obesity, the inclusion of faecal samples in future cohorts is warranted to elucidate the surgery’s impact on microbial composition and function. The impact on the metabolome on endoscopic MBS therapies, such as sleeve gastroplasty, will also be interesting to explore^42^.

## Conclusion

This systematic review and meta-analysis is the first to comprehensively evaluate the metabolic impacts of MBS in paediatric populations. Our findings reveal significant metabolic and anti-inflammatory benefits of MBS, with improvements in cardiovascular, glycaemic, and liver health markers. Importantly, these metabolic signatures in children parallel those observed in adult populations, underscoring the relevance and importance of early intervention, particularly given the evidence of obesogenic memory of fat tissue however the extent to which metabolic improvements are mediated by weight loss, hormonal shifts, or other mechanistic pathways remains to be elucidated. Importantly, while our study identifies associations between MBS and these outcomes, it does not establish causality. Further mechanistic studies are needed to differentiate weight-dependent from weight-independent drivers of metabolic benefit in this age group.

Despite these limitations, MBS remains a promising therapeutic intervention for adolescents with severe obesity who are at risk of long-term cardiometabolic complications. Outcomes from our review supports its role as a safe and effective tool within a multidisciplinary obesity management strategy.

## Supplementary Information

Supplementary Information.

## Data Availability

The data that support the findings of this study are available from the corresponding author, [A.T.M], upon reasonable request.

## Declarations

### Permission to reproduce material from other sources

No external materials other than what is already published was used in this article.
