# Supplementary material for: Impact of metabolic and bariatric surgery on the paediatric & adolescent metabolome: A systematic review and meta-analysis
Source: Sci Rep. 2025 Oct 16;15:36169. doi: 10.1038/s41598-025-20078-7 (PMC12533180; doi:10.1038/s41598-025-20078-7)
Supplement: Supplementary file 1 — Supplementary Information. [file 41598_2025_20078_MOESM1_ESM.docx]

**Supplementary File 1**

**Developing the search strategy**

The search strategy was required to capture weight and metabolic outcomes in paediatric or adolescent patients who underwent bariatric surgery using untargeted mass spectrometry methods. Review articles, conference abstracts, and animal and cell studies were excluded. The search strategy took account of the variability in the way authors describe their research and indexers index research with Medical Subject Headings (MeSH). All variations in spelling including truncated search terms using wild card characters and the “related articles” function were used in combination with the Boolean operators AND OR.

The following databases were searched:

- Ovid MEDLINE (R) 1946 to 2024 November 10;
- Ovid Embase 1974 to 2024 November 10;
- Cochrane library from inception to 2024 November 10

Records identified from each electronic and regional database were downloaded into a separate Endnote bibliographic database, which were then combined to generate a single large Endnote bibliography containing all the records identified from all the databases in which duplicate records were removed.

**Final search strategy**

The final search strategy and results for the MEDLINE search is shown below:

| Box 1 Exp | Explodes a MeSH to capture more specific MeSH. |
| --- | --- |
| / | Indicates that the search term is a Medical Subject Heading (MeSH). |
| AND | Achieves a Boolean AND Combination. |
| OR | Achieves a Boolean OR Combination. |
| .mp. | Searches for the term expressed in the title, original title, abstract, and subject heading. |
| * | Truncation operator, searches for words beginning with the stem, e.g. epidemiol* retrieves epidemiology, epidemiological and epidemiologic. |

**Medline search**

Embase Classic+Embase <1947 to 2024 November 10>

1 paediatric.mp. or exp pediatrics/ 275018

2 exp child/ or child.mp. 3976726

3 exp adolescent/ 2032510

4 1 or 2 or 3 4973603

5 bariatric surgery.mp. or exp obesity/ or exp bariatric surgery/ 771158

6 metabolic surgery.mp. 3350

7 5 or 6 771327

8 4 and 7 129265

9 metabolomic.mp. or exp metabolomics/ 83996

10 metabolome.mp. or exp metabolome/ 27637

11 9 or 10 95856

12 8 and 11 551
